# Characterization of Dendritic Cell and Regulatory T Cell Functions against *Mycobacterium tuberculosis* Infection

**DOI:** 10.1155/2013/402827

**Published:** 2013-05-23

**Authors:** Devin Morris, Brenda Gonzalez, Melissa Khurasany, Christine Kassissa, Jennifer Luong, Sarah Kasko, Shalin Pandya, Michael Chu, Po-Ting Chi, Steven Bui, Carlos Guerra, John Chan, Vishwanath Venketaraman

**Affiliations:** ^1^Department of Basic Medical Sciences, College of Osteopathic Medicine of the Pacific, Western University of Health Sciences, 309 East Second Street, Pomona, CA 91766-1854, USA; ^2^Department of Biological Sciences, California State Polytechnic University, 3801 West Temple Avenue, Pomona, CA 91768, USA; ^3^College of Dental Medicine, Western University of Health Sciences, 309 East Second Street, Pomona, CA 91766-1854, USA; ^4^College of Osteopathic Medicine of the Pacific, Western University of Health Sciences, 309 East Second Street, Pomona, CA 91766-1854, USA

## Abstract

Glutathione (GSH) is a tripeptide that regulates intracellular redox and other vital aspects of cellular functions. GSH plays a major role in enhancing the immune system. Dendritic cells (DCs) are potent antigen presenting cells that participate in both innate and acquired immune responses against microbial infections. Regulatory T cells (Tregs) play a significant role in immune homeostasis. In this study, we investigated the effects of GSH in enhancing the innate and adaptive immune functions of DCs against *Mycobacterium tuberculosis* (*M. tb*) infection. We also characterized the functions of the sub-populations of CD4+T cells such as Tregs and non-Tregs in modulating the ability of monocytes to control the intracellular *M. tb* infection. Our results indicate that GSH by its direct antimycobacterial activity inhibits the growth of intracellular *M. tb* inside DCs. GSH also increases the expressions of costimulatory molecules such as HLA-DR, CD80 and CD86 on the cell surface of DCs. Furthermore, GSH-enhanced DCs induced a higher level of T-cell proliferation. We also observed that enhancing the levels of GSH in Tregs resulted in downregulation in the levels of IL-10 and TGF-**β** and reduction in the fold growth of *M. tb* inside monocytes. Our studies demonstrate novel regulatory mechanisms that favor both innate and adaptive control of *M. tb* infection.

## 1. Introduction

*M. tb*, the causative agent for tuberculosis (TB), is a slow-growing, obligate aerobic bacterium, that is, transmissible through aerosolized droplets containing as few as 2-3 bacilli [1, 2]. Although appearing as a disease of past generations, *M. tb* infects one-third of the world's population [1, 2]. In this population, two billion people are asymptomatic carriers of the bacterial infection. According to WHO, eight million infected individuals will develop active TB and two million will die per year [3]. Of the two billion individuals latently infected with *M. tb*, 10% will undergo reactivation of *M. tb* infection leading to active disease whenever their immune system gets compromised due to ageing, corticosteroid treatment, or most commonly, coinfection with human immunodeficiency virus (HIV) [4]. *M. tb* can resist complete clearance by the host immune system due to several factors including its ability to persist and remain in a dormant state in antigen presenting cells (APCs) for a lengthy duration of time [3]. 

 GSH is a tripeptide that regulates intracellular redox and other important aspects of cell physiology [5]. GSH plays a major role in enhancing the functions of immune cells. GSH is essential for cellular homeostasis and plays a vital role in diverse cellular functions. GSH synthesis occurs within cells in two closely linked, enzymatically controlled reactions with the availability of cysteine usually being the rate-limiting factor [5]. In healthy cells more than 90% of the total GSH pool is in the reduced form (GSH or *r*GSH) and less than 10% exists in the oxidized disulfide form (GSSG). Among the two forms, *r*GSH is considered to be the functional form having the antioxidant and other immune enhancing properties [6]. 

The two major layers of defense against *M. tb* infection include preformed innate immunity that lacks specificity and a highly specific and effective adaptive immunity. DCs are potent APCs, linking the innate and adaptive immune responses [7]. DCs have the unique ability to migrate from the site of infection to a draining lymph node and subsequently recruit T cells to the site of infection thereby effectively activating the acquired immune response [8]. In addition, DCs carry a myriad of functions ranging from influencing different lymphocytes such as B cells, natural killer (NK) cells, and natural killer T (NKT) cells to initiating different T lymphocyte responses such as Th1/Th2, regulatory T cells and peripheral T cell deletion [8].

 Regulatory T cells (Tregs) are critical for the maintenance of immune cell homeostasis as evidenced by the catastrophic consequences of genetic or physical ablation of the Treg population [9]. Specifically, Treg cells maintain order in the immune system by enforcing a dominant negative regulation on other immune cells [9]. 

 In this study, we tested the effects of GSH in regulating the functions of DCs to control *M. tb* infection by performing *in vitro* studies using isolated cells from healthy subjects. The production of IL-10 and IL-12 from DCs was also analyzed to indirectly elucidate whether a cell-mediated immunity (IL-12) or an antibody-mediated (IL-10) response is produced when DCs were treated with GSH-enhancing agents such as N-acetyl cysteine (NAC, a GSH precursor) and liposomal glutathione (L-GSH). Increased production of IL-12 accompanied by Th1 CD4 T-cell responses is considered crucial for controlling *M. tb* infection. We also examined the ability of GSH to enhance the expressions of costimulatory molecules on the cell surface of DCs and to induce proliferation of T-cells. 

Additionally, we characterized the effects of GSH in modulating the functions of both Tregs and non-T regs (sub-populations of CD4+ T cells) to control *M. tb* infection inside monocytes. Finally, we investigated whether increasing GSH concentrations in Tregs would result in downregulation of TGF-*β* and IL-10 levels leading to improved control of *M. tb* infection inside monocytes. 

 Considering DCs crucial role in recruiting T cells to aid in infection, we show that by increasing the intracellular levels of GSH, DC performance is enhanced in its innate function of inhibiting the intracellular growth of *M. tb* as well as its adaptive immune role as professional APCs. Furthermore, we also show that enhancing GSH in Tregs resulted in downregulation in the levels of TGF-*β* and IL-10 leading to better control of *M. tb* infection inside monocytes. Our results signify the importance of GSH in enhancing both the innate and adaptive immune responses against *M. tb* infection.

## 2. Materials and Methods

### 2.1. Statement of Ethics

All studies were approved by both the Institutional Review Board and the Institutional biosafety committee of the Western University of Health Sciences. All study participants were above the legal age of consent at the time of participation and written informed consent was obtained from all volunteers prior to participation in the study. 

### 2.2. Subjects

Healthy subjects without HIV infection or a history of TB were recruited from the faculty and staff of Western University of Health Sciences. Thirty-five milliliters (mL) of blood was drawn once from both healthy volunteers after obtaining signed informed consent. 

### 2.3. Isolation of Monocytes and *In Vitro* Culture for Differentiation into DCs

Peripheral blood mononuclear cells (PBMCs) were isolated from the whole blood of healthy individuals by density gradient centrifugation using ficoll histopaque, a high density pH neutral polysaccharide solution (Sigma). Monocytes were isolated from PBMCs as follows: PBMCs (1 × 10^5^ cells/well) were distributed in poly-L lysine (0.005%, Electron Microscopy Sciences) treated 96-well tissue culture plates, and incubated overnight at 37°C to facilitate monocyte adherence. Following overnight adherence, the nonadherent cells were removed and fresh Roswell Park Memorial Institute media (RPMI (Sigma)) supplemented with 5% human AB serum (Sigma), 2 mM glutamine, 20 ng/mL GM-CSF (Ebioscience), and 40 ng/mL IL-4 (Ebioscience) was added to the adherent monocytes which were then incubated for 7 days to allow monocytes to differentiate into DCs. 

### 2.4. Preparation of Bacterial Cells for DC Infection

All infection studies were performed using the virulent laboratory strain of *M. tb*, H37Rv inside the biosafety level 3 (BSL-3) facility. *M. tb* was processed for infection as follows: static cultures of H37Rv at their peak logarithmic phase of growth (between 0.5 and 0.8 at A600) were used for infection of DCs. The bacterial suspension was washed and resuspended in RPMI (Sigma) containing AB serum (Sigma). Bacterial clumps were disaggregated by vortexing five times with 3 mm sterile glass beads. The bacterial suspension was passed through a 5 *μ*m syringe filter (Millipore) to remove any existing clumps. The total number of organisms in the suspension was ascertained by plating. Processed H37Rv was frozen as stocks at −80°C. At the time of infection, frozen stocks of processed H37Rv were thawed and used for infection of DCs and monocytes.

### 2.5. Assay of GSH Levels in DCs from Healthy Subjects

We determined the effects of *M. tb* infection in decreasing the levels of total GSH in isolated DCs from healthy subjects by spectrophotometry using an assay kit from Arbor Assays. Furthermore, we also tested the ability of GSH-enhancing agents such as NAC and L-GSH in restoring the levels of total GSH and the ratio of *r*GSH versus GSSG in *M. tb*-infected DCs. Monocyte derived DCs were infected with processed H37Rv at a multiplicity of infection of 1 : 1 (1 bacterium for every single DC) and incubated for 2 hours to allow for phagocytosis. Unphagocytosed bacteria were removed by washing the infected DCs three times with warm sterile PBS. Infected DCs were cultured in RPMI + 5% AB serum at 37°C + 5% CO_2_ in the presence and absence of NAC (10 and 20 mM) or L-GSH (10 and 20 *μ*M, Your Energy Systems, LLC Palo Alto, CA, USA. The liposomal formulation of GSH (L-GSH) used in these studies is a proprietary product of Your Energy Systems (YES), LLC (Palo Alto, CA, USA) called ReadiSorb. Infected DCs were terminated at 5 days after infection to determine the intracellular levels of GSH. Uninfected DCs maintained in culture for 5 days served as controls for baseline levels of GSH. Briefly, DCs (3 × 10^5^) were washed and resuspended in ice cold 5% 5-sulfo-salicylic acid dehydrate solution (MP Biomedicals). Supernatants collected after centrifugations were analyzed for total GSH and oxidized GSH (GSSG) as per manufacturer's instructions. *r*GSH was calculated by subtracting measured GSSG concentrations from the measured total GSH concentrations. All GSH measurements were normalized with total protein levels. 

### 2.6. Assay of Total Protein Levels in Lysates

Proteins in the isolated cell lysates were measured by Bradford's method [10] using a Coomassie protein assay reagent (Thermo Scientific). This assay helped to determine the amount of protein in each well. By dividing the total protein value from the GSH assay, it helped to confirm that the variation of GSH is due to the treatments added.

### 2.7. Intracellular Survival of H37Rv in GSH-Enhanced DCs

To examine the role of DCs and GSH in innate defense against *M. tb* infection, we determined the intracellular survival of *M. tb* inside DCs that were cultured in the presence and absence of GSH-enhancing agents. DCs were infected with processed H37Rv at a low dose multiplicity of infection of 1 : 10 (1 bacterium for every 10 DCs) and incubated for 2 hours to allow for phagocytosis. Unphagocytosed mycobacteria were removed by washing the infected DCs three times with warm sterile PBS. Infected DCs were cultured in RPMI + 5% AB serum at 37°C + 5% CO_2_ in the presence and absence of NAC (5, 10, and 20 mM), L-GSH (5, 10, and 20 *μ*M, Your Energy Systems, LLC Palo Alto, CA, USA), or buthionine sulfoximine (BSO-500 *μ*M). BSO inhibits the synthesis of GSH by inhibiting the activity of the rate limiting step enzyme that is involved in the synthesis of GSH leading to decreased intracellular levels of GSH. H37Rv-infected DCs that were sham-treated (RPMI + 5% AB serum) served as a control. Infected DCs were terminated at 1 hour and 5 days after infection to determine the intracellular survival of H37Rv. 

### 2.8. Termination of H37Rv-Infected DC Cultures and Measurement of Colony Forming Units (CFUs)

Termination of H37Rv*-*infected DCs was performed by the addition of 200 *μ*L of sterile distilled water to each culture well. The collected DC lysates were diluted in sterile water and plated on 7H11 medium (Hi Media) enriched with albumin dextrose complex (ADC), to estimate the extent of H37Rv growth in DCs by counting the CFUs.

### 2.9. Assay of IL-1, IL-12, and IL-10 in DC Supernatants

Levels of IL-1, IL-12 and IL-10 in DC supernatants were measured by enzyme linked immunosorbent assay (ELISA) (eBioscience). 

### 2.10. Immunocytochemical Analysis of Costimulatory Markers Expressions on the Cell Surface of DCs

The effects of GSH-enhancing agents in augmenting the expressions of costimulatory molecules on the cell surface of DCs were determined by immuno-fluorescent staining. DCs (both uninfected (control category) and H37Rv-infected) were incubated for 5 days on sterile glass cover slips in the presence and absence of GSH-enhancing agents. DCs were fixed with 3.8% paraformaldehyde (PFA) in phosphate buffered saline (PBS) for 30 minutes. Fixed DCs were washed twice for 5 minutes in ice cold PBS and then blocked for 30 minutes in 1% bovine serum albumin (BSA) in PBS + 0.2% Tween-20 (PBST). Blocked DCs were incubated with mouse anti-human HLA-DR IgG, mouse anti-human CD80 IgG, and mouse anti-human CD86 IgG (1 *μ*g/mL each, Ebioscience) in PBST, overnight at 4°C with mild shaking. Antibodies against HLA-DR and CD80 were labeled with FITC 488 (green) whereas antibodies against CD86 were labeled with PE 546 (red). After incubation with fluorescent labeled antibodies, stained DCs were washed three times for 5 minutes with PBS to remove excess stain. A single drop of mounting media containing 4′, 6-diamidino-2-phenylindole (DAPI) was placed on glass slides before inverting the glass cover slips with attached, stained DCs and placing them on the slide. The cover slips were then sealed to the glass slide using nail polish around the edges of the cover slip. The stained slides were then viewed using an inverted fluorescent microscope. Images were obtained using an integrated digital camera. Images were subsequently analyzed using ImageJ, a free software program available from the National Institutes of Health (http://rsbweb.nih.gov/ij/). Correcting for background fluorescence, average fluorescent intensity was measured for each labeled protein.

### 2.11. Intracellular Survival of H37Rv in Matured DCs

Lipopolysaccharide (LPS), an activating agent is also known to induce maturation of DCs. We therefore tested the effects of GSH in improving the control of *M. tb* infection inside mature DCs by determining the intracellular survival of H37Rv in GSH-enhanced and LPS-treated DCs. Monocyte derived DCs were infected with processed H37Rv at a multiplicity of infection of 1 : 1 (1 bacterium for every single DC) and incubated for 2 hours to allow for phagocytosis. Unphagocytosed mycobacteria were removed by washing and infected DCs were cultured in medium containing LPS (1 *μ*g/mL) in the presence and absence of NAC (5, 10, and 20 mM), L-GSH (5, 10, and 20 *μ*M), or BSO (500 *μ*M). Infected DCs were terminated at 1 hour and 5 days after infection to determine the intracellular survival of H37Rv in mature DCs. H37Rv-infected + LPS-treated DCs served as controls for GSH-enhancing agent treatment groups.

### 2.12. DCs and T Cell Proliferation Assays

We determined the ability of GSH-enhanced DCs to induce proliferation of T cells by quantifying the fluorescence intensity of carboxyfluorescein diacetate, succinimidyl ester (CFSE). DCs were treated overnight with NAC (10 mM) either alone or in combination with NAC + LPS (1 *μ*g/mL). Following overnight incubation with NAC, DCs were infected with H37Rv, washed, and resuspended in fresh media containing no additives. Allogeneic T cells were isolated from PBMCs using a nylon wool column (Polysciences). Isolated T cells were washed two times in PBS to remove any serum. T cells were resuspended in PBS at a cell density of 5–10 × 10^6^/mL. CFSE, at a concentration of 1 *μ*M, was added to allogeneic T cell suspension, mixed immediately and incubated for 10 minutes at room temperature in the dark. Labeling was stopped by the addition of 4-5 volumes of cold complete media followed by incubation on ice for 5 minutes. T cells were then washed three times with complete media and evenly distributed to the wells containing infected DCs. CFSE allows for cell division tracking of T cells, that is, the tracking of the proliferation of T cells upon the coincubation with H37Rv-infected and NAC treated DCs. In separate studies, GSH-levels in T cells (not DCs) were manipulated by overnight treatment with different concentrations of NAC (5, 10 and 20 mM). Following overnight incubation with NAC, T cells were washed three times with PBS, labeled with CFSE and then added to the infected DCs to determine the ability of GSH-enhanced T cells to effectively respond to the *M. tb*-infected DCs and proliferate. Seven days after incubation, T cells from both the studies (GSH-altered DCs and GSH-altered T cells) were aspirated and fixed in PFA and analyzed for T cell proliferation using flow cytometry outside of BSL-3 lab.

### 2.13. Determination of Intracellular Viability of H37Rv in Cocultures of Infected Monocytes and CD4+ T Cells (Non-T regs and Tregs)

We characterized the roles of sub-populations of CD4+ T cells, specifically Tregs (CD4+CD25+ T-cells) and non-T regs (CD4+CD25− T cells), in regulating the host immune responses against *M. tb* infection by quantifying the intracellular viability of H37Rv inside monocytes that were cultured in the presence and absence of Tregs and non-Tregs. Tregs and non-Tregs were isolated from PBMCs derived from healthy subjects using midi-MACS LD columns and mini-MACS MS columns from Miltenyi Biotech. This method of isolation resulted in 99% pure population of Tregs and non-Tregs [11]. Adherent monocytes were infected with processed H37Rv at a multiplicity of infection of 1 : 1 and incubated for 2 hours for phagocytosis. Unphagocytosed mycobacteria were removed by washing the infected monocytes three times with sterile PBS. Infected monocytes were cultured in RPMI containing 5% AB serum in presence and absence of autologous Tregs and non-Treg cells. Prior to co-incubation with infected-monocytes, autologous CD4+ T cells (both Tregs and non-Tregs) were incubated overnight with NAC (10 mM), washed with PBS, resuspended in fresh RPMI containing AB serum (without any stimulants), and then added to the infected monocytes (monocyte: T cell ratio was adjusted to 1 : 1). H37Rv-infected monocytes cultured in the absence of CD4+ T cells (Tregs and non-Tregs) served as controls. Infected monocyte T cell cocultures were terminated at 1 hour and 5 days after infection to determine the intracellular survival of H37Rv. 

### 2.14. Termination of *M. tb*-Infected Monocytes-Autologous CD4+ T Cell Cocultures

H37Rv-infected monocytes cultured in the presence and absence of Tregs and non Tregs were terminated at 1 hour and 5 days after infection. During termination, supernatants were removed and adherent monocytes were lysed by addition of 200 *μ*L sterile cold distilled water. 25 *μ*L of 10-fold diluted lysates were plated on 7H11 medium (Hi Media) enriched with ADC, to estimate the extent of H37Rv growth or killing in co-cultures of monocytes and CD4+ T cells. Cell-free supernatants were plated to determine extracellular H37Rv growth. Cell-free supernatants were also used for determining the levels of IL-10, and TGF-*β*.

### 2.15. Assay of IL-10 and TGF-*β* in Supernatants from Cocultures of Monocytes and CD4+ T Cells

Cytokines (IL-10 and TGF-*β*) were measured in supernatants from co-cultures of monocytes and CD4+ T cells (Tregs and non Tregs) by ELISA (eBioscience). 

### 2.16. Statistical Analysis

All statistical analysis was done using GraphPad Prism6 software on the mean ± standard error for *n* = 5 individuals, unless otherwise indicated. Results were considered significant for *P* ≤ 0.05.

## 3. Results

### 3.1. GSH Measurement in Lysates from DCs

**Figure 1:**
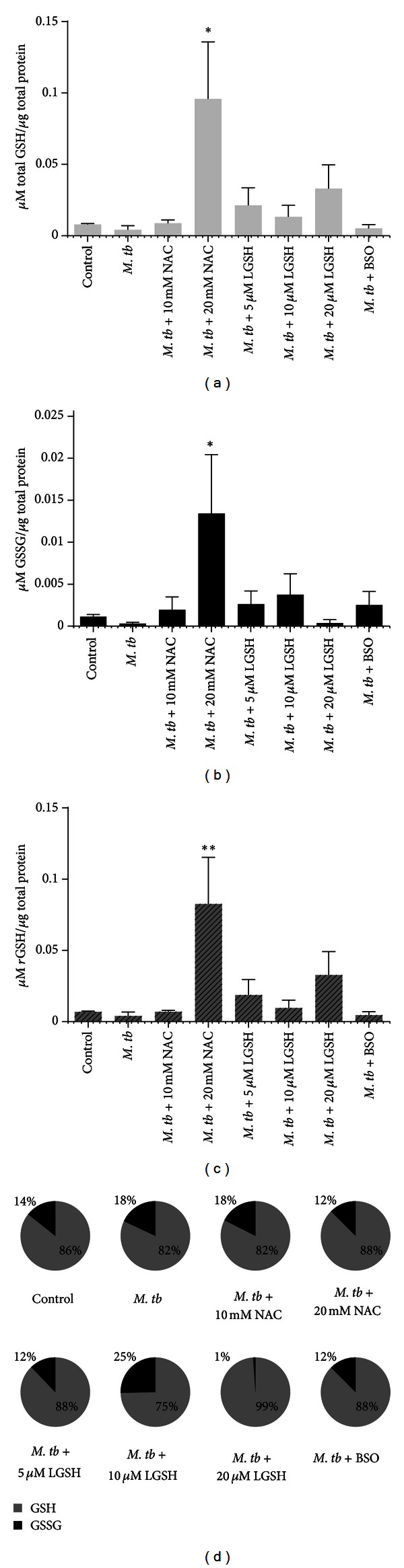
GSH measurements in DCs infected with H37Rv and treated with NAC, L-GSH, or BSO. GSH levels were measured in isolated DCs from healthy subjects that were infected *in vitro *with H37Rv and treated with various stimulants such as NAC, L-GSH, and BSO, by spectrophotometry using an assay kit from Arbor Assays. All GSH measurements were normalized with total protein levels. Proteins in the cell lysates of DCs were measured by Bradford's method using a Coomassie protein assay reagent. Results shown are for *n* = 3 individuals and analyzed for significance by ANOVA, Dunnett's multiple comparisons test, comparing all treatment categories to the infected-untreated control. (a) Total GSH (*r*GSH  +  GSSG), *P ≤ 0.05. (b) GSSG, *P ≤ 0.05. (c) *r*GSH was calculated by subtracting measured GSSG concentrations from the measured total GSH concentrations, **P ≤ 0.01. (d) Composition of total GSH, represented in percentages of *r*GSH and GSSG.

Infection of DCs with H37Rv resulted in 50% decrease in the intracellular levels of total GSH compared to uninfected DCs (Figure 1(a)). Treatment of H37Rv-infected DCs with 10 mM NAC resulted in restoration of GSH to similar levels as uninfected DCs (Figure 1(a)). Maximum enhancement in the intracellular levels of both total and *r*GSH was observed when H37Rv-infected DCs were treated with 20 mM NAC (8-fold increase) or 20 *μ*M L-GSH (3-fold increase) (Figures 1(a), 1(b), 1(c) and 1(d)). We also compared the percentage of GGSG and *r*GSH for various treatment conditions and we observed that treatment of H37Rv-infected DCs with 20 *μ*M L-GSH resulted in 99% *r*GSH and just 1% GSSG highlighting the effective restorative effects of L-GSH at 1000-fold lower concentration compared to NAC.

### 3.2. Intracellular Survival of H37Rv in DCs

**Figure 2:**
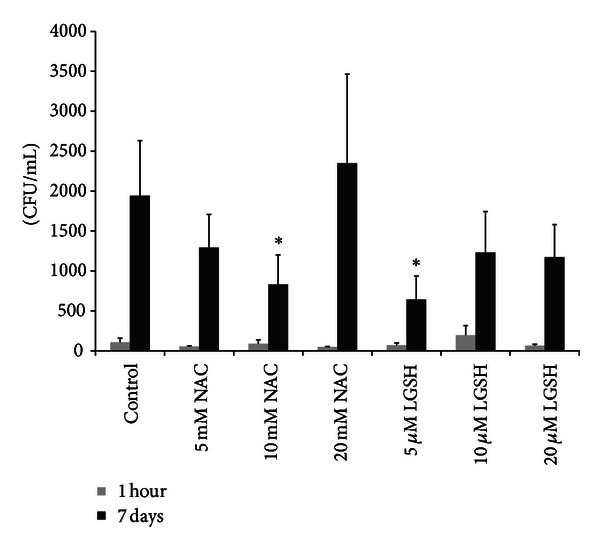
Intracellular survival of H37Rv in DCs treated with NAC and L-GSH. Lysates from H37Rv infected DCs were plated on 7H11 agar and colonies were counted. Results were analyzed by ANOVA with Dunnett's multiple comparisons test, comparing all treatment categories to the infected-untreated control, *P ≤ 0.05.

In our low-dose *M. tb* infection studies, we observed a twentyfold increase in the intracellular growth of H37Rv in unstimulated DCs between the initial and final time points of termination (Figure 2). Treatment of DCs with GSH-enhancing agents such as NAC (5 mM and 10 mM) or L-GSH (5 *μ*M, 10 *μ*M, and 20 *μ*M) resulted in reduction in the fold increase in the growth of *M. tb* inside DCs (Figure 2). Among various concentration of NAC that were tested, maximum reduction in the fold-growth of *M. tb* was observed when DCs were treated with 5 mM NAC (only tenfold increase in the growth of H37Rv compared to unstimulated DCs in which there was twentyfold increase in the growth of *M. tb*). Similarly, treatment of H37Rv-infected DCs with 5 *μ*M concentration of L-GSH (Figure 2) resulted in utmost reduction in the fold-growth of H37Rv (there was only ninefold increase in the intracellular growth of H37Rv between the initial and final time point of termination). Although treatment with 20 mM NAC resulted in maximum enhancement in the intracellular levels of GSH in DCs, we did not however observe any decrease in the fold growth of *M. tb* under these conditions indicating the need for maintaining optimum levels of GSH in the intracellular environment in order to effectively inhibit the growth of *M. tb* (Figure 2).

### 3.3. IL-1 Assay in DC Supernatants

**Figure 3:**
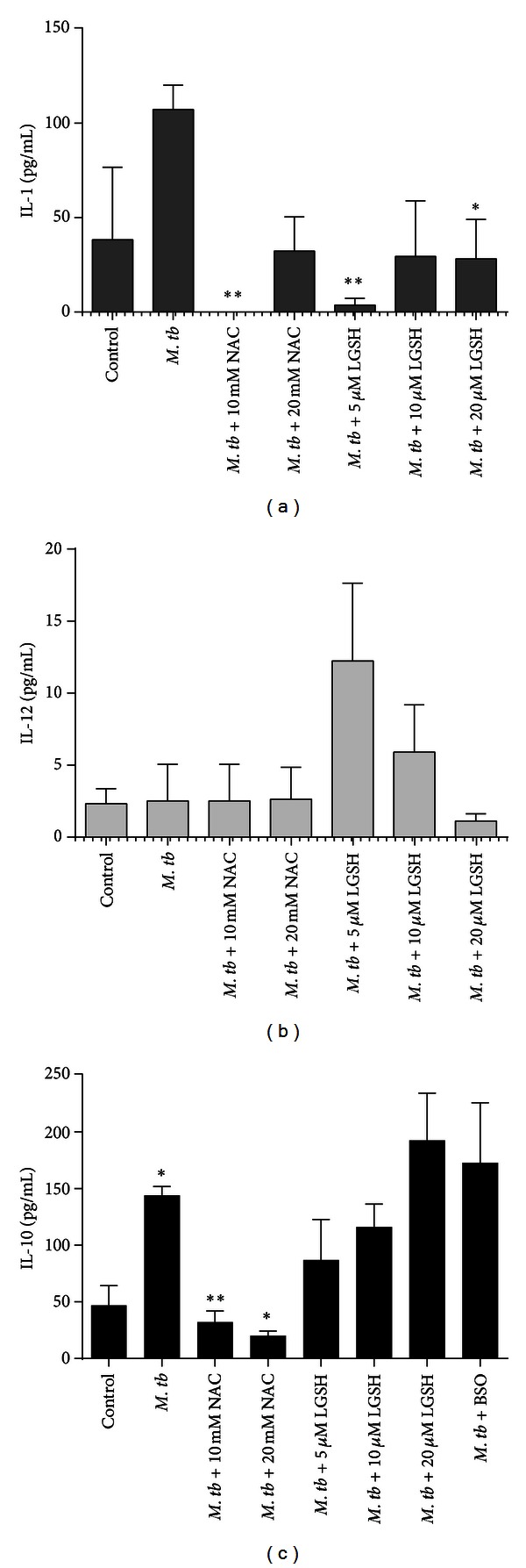
Cytokine measurements performed in supernatants derived from H37Rv-infected DCs. Cytokines in DC supernatants were measured on *n* = 3 samples by ELISA. Results were analyzed by ANOVA with Dunnett's multiple comparisons test, comparing all treatment categories to the infected-untreated control. Infected-untreated controls were compared to the untreated-uninfected control using the Student's *t*-test, *P ≤ 0.05, **P ≤ 0.01. (a) IL-1. (b) IL-12. (c) IL-10.

H37Rv infection of DCs resulted in 2.5-fold increase in the production of IL-1 compared to uninfected DCs (Figure 3(a)). Enhancing the levels of GSH in DCs caused decrease in the production of IL-1. Specifically, treatment of DCs with 10 mM NAC resulted in undetectable levels of IL-1 (Figure 3(a)). Similarly treatment of H37Rv-infected DCs with 5 *μ*M L-GSH resulted in a notable decrease in the levels of IL-1 compared to other concentrations of L-GSH used in the treatment of H37Rv-infected DCs (Figure 3(a)).

### 3.4. Assay of IL-12 and IL-10 in Supernatants from DCs

Treatment of H37Rv-infected DCs with L-GSH (5 *μ*M) resulted in increased production of IL-12, a Th1 polarizing cytokine (Figure 3(b)). H37Rv infection of DCs resulted in 3-fold increase in the production of IL-10 compared to uninfected DCs (Figure 3(c)). Treatment of H37Rv-infected DCs with either NAC (10 mM) or L-GSH (5 *μ*M) resulted in decreased production of IL-10, a Th2 polarizing cytokine (Figure 3(c)). Decreased IL-10 may favor a Th1 response.

### 3.5. Assay of DC Cell Surface Markers

**Figure 4:**
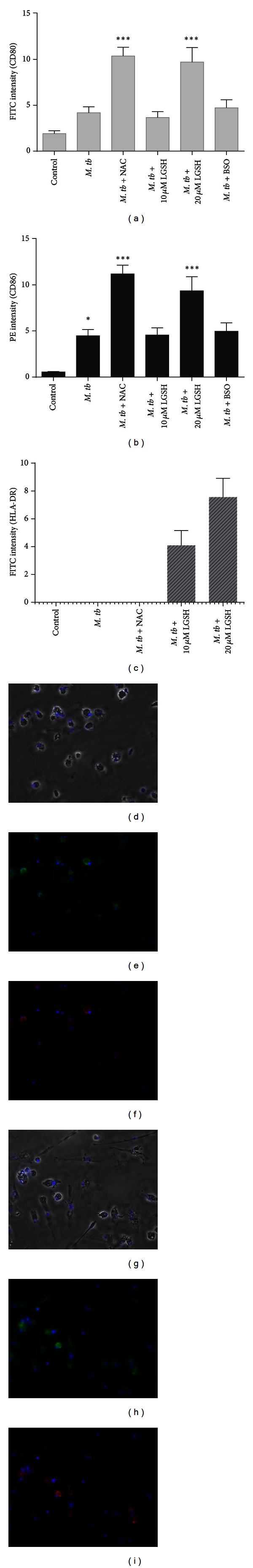
Immunocytochemical analysis of costimulatory marker expressions on the cell surface of DCs. Co-stimulatory marker expression on the surface of DCs was measured by immuno-cytochemistry. Results were analyzed by ANOVA with Dunnett's multiple comparisons test, comparing all treatment categories to the infected-untreated control. Infected-untreated controls were compared to the untreated-uninfected control using the Student's *t*-test, *P ≤ 0.05, ***P ≤ 0.001. (a) Quantification of CD80 expression on DCs. (b) Quantification of CD86 expression on DCs. (c) Quantification of HLA-DR expression on DCs. (d)–(i) Fluorescent microscopy images of DCs from control and NAC-treated categories (20x magnification). Antibodies against HLA-DR and CD86 are labeled with FITC 488 (green) and PE 546 (red), respectively. (d) Bright field view of control DCs overlaid with fluorescent microscopy images using DAPI. (e) Control category DCs labeled with FITC conjugated anti- HLA-DR antibodies. (f) Control category DCs labeled with PE conjugated anti-CD-86 antibodies. (g) Bright field view of NAC treated DCs overlaid with fluorescent microscopy images using DAPI. (h) NAC treated DCs labeled with FITC conjugated anti- HLA-DR antibodies. (i) NAC treated DCs labeled with PE conjugated anti-CD-86 antibodies.

We examined the effects of GSH in increasing the expressions of costimulatory molecules on the cell surface *M. tb*-infected DCs by immunofluorescence staining. H37Rv infection of DCs resulted in 2-fold increase in the expression of CD80 compared to uninfected control DCs. We observed that treatment of H37Rv-infected DCs with 10 mM NAC and 20 *μ*M L-GSH resulted in 2.5-fold increase in the expression of CD80 compared to H37Rv-infected DCs and 5-fold increase in comparison to uninfected control category. Consistent with our observations on the CD80 marker, we also found a similar trend with the expression of CD86 marker; that is, treatment of H37Rv-infected DCs with 10 mM NAC and 20 *μ*M L-GSH resulted in 2.5-fold increase in the expression of CD86 compared to H37Rv-infected DCs and 5-fold increase in comparison to uninfected control. Importantly, L-GSH added at 500x lower concentration compared to NAC is still able to induce the upregulation of both CD80 and CD86 molecules (Figures 4(a) and 4(b)). We observed a noticeable increase in the expression of HLA-DR only when the H37Rv-infected DCs were treated with 10 *μ*M L-GSH (4-fold increase) and 20 *μ*M L-GSH (5-fold increase) (Figure 4(c)). Our results signify the importance of GSH in enhancing the expressions of HLA-DR, CD80 and CD86 on the cell surface of *M. tb*-infected DCs.

### 3.6. Determination of Intracellular Viability of H37Rv in LPS-Treated DCs

**Figure 5:**
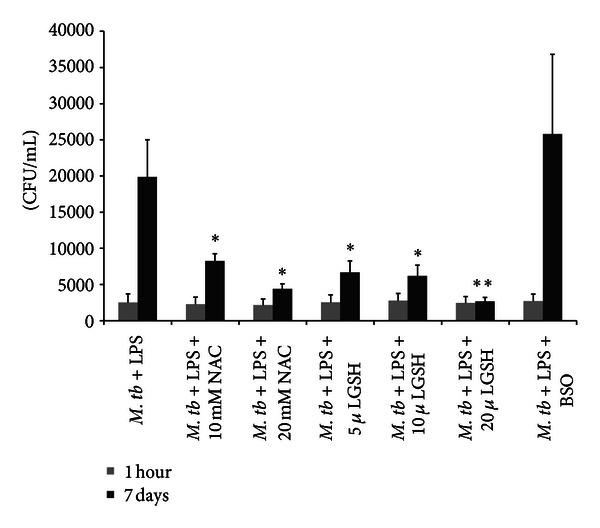
Intracellular survival of H37Rv inside mature DCs. Monocyte derived DCs were infected with processed H37Rv at a multiplicity of infection of 1 : 1 (1 bacterium per DC). Infected DCs were cultured in medium containing LPS (1 *μ*g/mL) in the presence and absence of NAC (5, 10, and 20 mM), L-GSH (5, 10, and 20 *μ*M), or BSO (500 *μ*M). Infected DCs were terminated at 1 hour and 5 days after infection to determine the intracellular survival of H37Rv in mature DCs. Results were analyzed using one-way ANOVA with Dunnett's multiple comparisons test, comparing all treatment categories to the infected-LPS treated control, *P ≤ 0.05, **P ≤ 0.01.

We tested the effects of GSH-enhancing agents (NAC/L-GSH) in improving the control of *M. tb* inside matured DCs. Maturation of DCs was induced by treatment with LPS. We observed an 8-fold increase in the intracellular growth of H37Rv in LPS-treated DCs between the initial and final time point of termination (Figure 5). Treatment of H37Rv-infected DCs with a combination of LPS + NAC (20 mM) resulted in effective inhibition in the growth of intracellular H37Rv (there was only 2-fold increase in the growth of H37Rv). Treatment of H37Rv-infected DCs with a combination of LPS + L-GSH (20 *μ*M) resulted in complete stasis in the growth of H37Rv (Figure 5). Treatment of infected DCs with BSO (a GSH synthesis inhibitor) resulted in 10-fold increase in the intracellular growth of H37Rv in DCs (Figure 5). Our results indicate that treatment of *M. tb*-infected DCs with a combination of NAC + LPS resulted in further improvement in the control of *M. tb* infection. Additionally, treatment of *M. tb*-infected DCs with a combination of L-GSH + LPS resulted in complete inhibition in the growth of intracellular *M. tb*.

### 3.7. Determination of Allogeneic T Cell Proliferation in Response to H37Rv-Infected DCs

**Figure 6:**
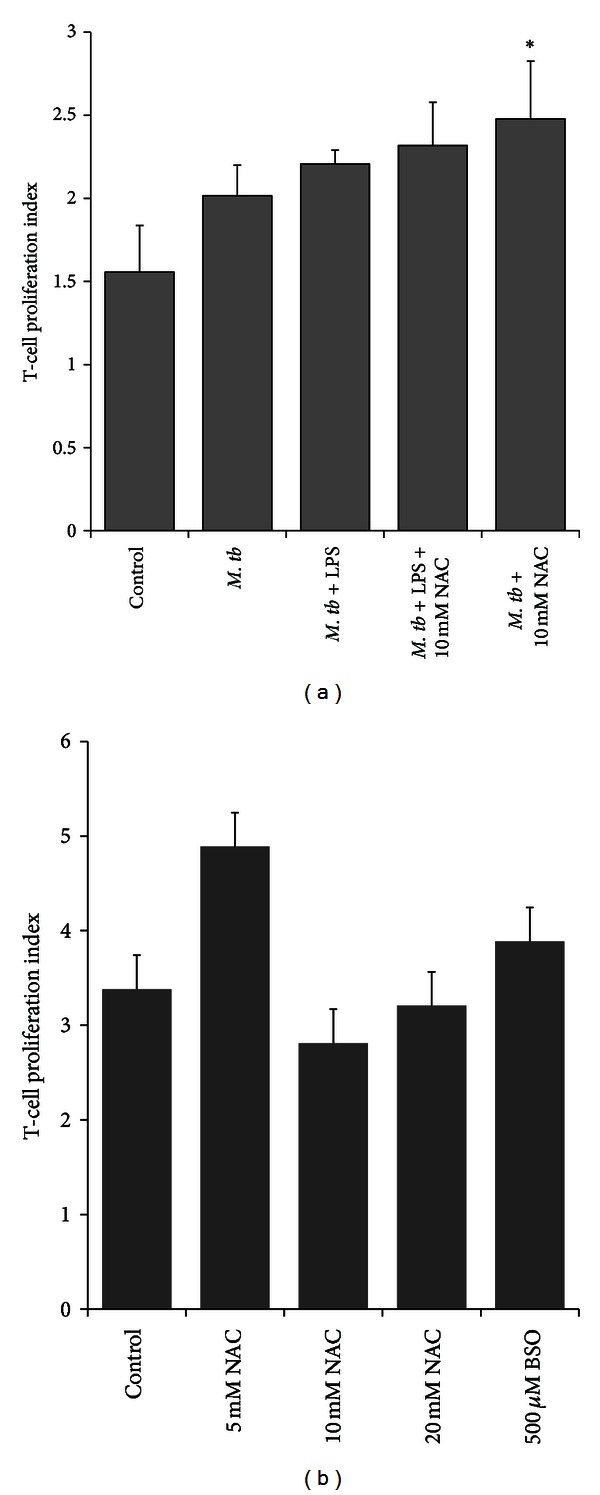
DCs and T cell proliferation assays. (a) Allogeneic T cell proliferation in response to H37Rv-infected, NAC treated DCs. DCs were treated overnight with NAC (10 mM) either alone or in combination with NAC  +  LPS (1 *μ*g/mL). Following overnight incubation with NAC, DCs were infected with H37Rv, washed, and resuspended in fresh media containing no additives. Allogeneic T cells isolated from PBMCs using a nylon wool column were stained with CFSE (1 *μ*M). Labeled T cells were added to the wells containing infected DCs. Seven days post-incubation, T cells were aspirated and fixed in PFA and analyzed for T cell proliferation using flow cytometry. (b) Proliferation of GSH-enhanced T cells in response to H37Rv-infected DCs. GSH levels in T cells (not DCs) were manipulated by overnight treatment with different concentrations of NAC (5, 10, and 20 mM). Following overnight incubation with NAC, T cells were washed three times with PBS, labeled with CFSE, and then added to the infected DCs to determine the ability of GSH-enhanced T cells to effectively respond to the *M. tb*-infected DCs and proliferate. Seven days after incubation, T cells were aspirated and fixed in PFA and analyzed for T cell proliferation using flow cytometry.

We assessed the effects of GSH-enhancement in *M. tb*-infected DCs to improve their ability to induce proliferation of allogeneic T cells by CFSE staining. We observed that treatment of H37Rv-infected DCs with NAC (10 mM) resulted in a maximum increase in the proliferation index of allogeneic T-cells (Figure 6(a)). Treatment of H37Rv-infected DCs with a combination of LPS + NAC did not result in an additional increase in proliferation index of T cells. These results demonstrate the effects of GSH in enhancing the ability of *M. tb*-infected DCs to induce proliferation of T cells (Figure 6(a)). 

### 3.8. Determination of T Cell Proliferation of GSH-Altered T Cells in Response to H37Rv-Infected DCs

We then investigated the proliferation capacity of GSH-enriched allogeneic T cells in response to *M. tb*-infected DCs. Our results indicate that treatment of allogeneic T cells with 5 mM NAC showed the highest T-cell proliferation index. 10 mM and 20 mM treated T-cells did not result in an increase in the proliferation index (Figure 6(b)). 

### 3.9. Determination of Intracellular Viability of H37Rv in Co-Cultures of H37Rv-Infected Monocytes and Non-Tregs

**Figure 7:**
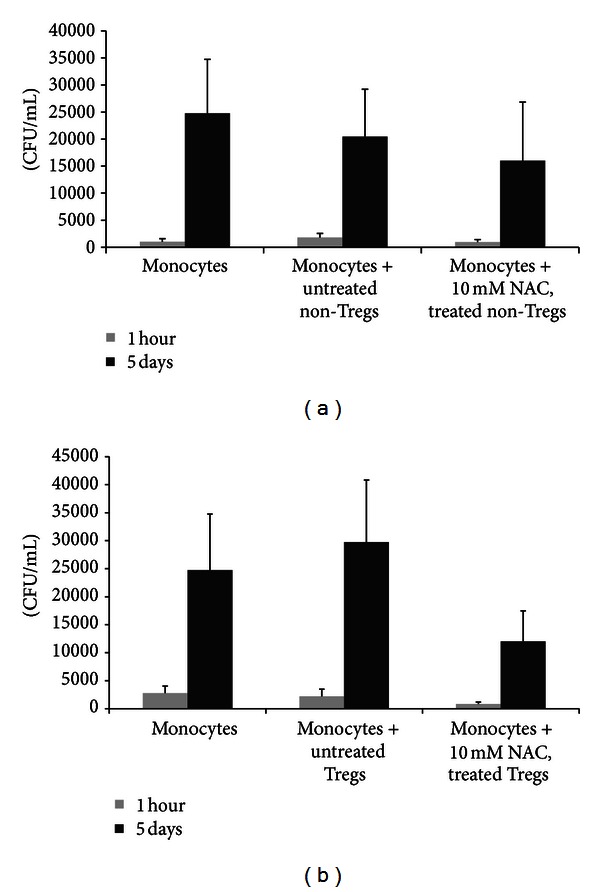
Determination of intracellular viability of H37Rv in cocultures of H37Rv-infected monocytes and CD4+ T cells (non-Tregs and Tregs). Tregs and non-Tregs were isolated from PBMCs derived from healthy subjects using midi-MACS LD columns and mini-MACS MS columns from Miltenyi Biotech. Adherent monocytes were infected with processed H37Rv at a multiplicity of infection of 1 : 1 and incubated for 2 hours for phagocytosis. Unphagocytosed mycobacteria were removed by washing the infected monocytes three times with sterile PBS. Infected monocytes were cultured in RPMI containing 5% AB serum in presence and absence of autologous non-Tregs (a) and Tregs cells (b). Prior to co-incubation with infected monocytes, autologous CD4+ T cells (both Tregs and non-Tregs) were incubated overnight with NAC (10 mM), washed with PBS, re-suspended in fresh RPMI containing AB serum (without any additives), and then added to the infected monocytes (monocyte: T cell ratio was adjusted to 1 : 1). Infected monocyte-CD4+ T cell cocultures were terminated at 1 hour and 5 days after infection to determine the intracellular survival of H37Rv. Infected monocytes cultured in the absence of T cells served as negative controls. Results were analyzed using one-way ANOVA with Dunnett's multiple comparisons test, comparing all T-cell co-culture categories to the monocyte only control. Our results did not achieve statistical significance.

We observed a 25-fold increase in the growth of H37Rv inside human monocytes. Co-culture of H37Rv-infected monocytes with non-Treg population (CD4 T cells minus the Treg population that is, CD4+CD25− T-cells) resulted in reduction in the fold growth of H37Rv inside monocytes. This underscores the importance of CD4+CD25− non-T reg population in augmenting the capacity of monocytes to control *M. tb* infection. Treatment of non-T reg population with NAC resulted in further reduction in the CFU counts of H37Rv at 5 day time point of termination indicating improved control of *M. tb* infection when monocytes were cultured in the presence of NAC-treated non-Treg cells (Figure 7(a)).

### 3.10. Determination of Intracellular Viability of H37Rv in Co-Cultures of H37Rv-Infected Monocytes and Tregs

We characterized the role of Tregs in *M. tb* infection by determining the intracellular survival of H37Rv in monocytes cultured in the presence and absence of Tregs. We observed a 20-fold increase in the growth of H37Rv in monocytes that were cultured in the absence of T cells. In the presence of Tregs there was further increase in the fold growth of H37Rv inside monocytes. NAC treatment of Tregs resulted in only 12-fold increase in the intracellular growth of H37Rv (Figure 7(b)). In fact, the fold increase in the survival of H37Rv in co-cultures monocytes + NAC-treated Tregs was lowest compared to other treatment groups such as monocytes alone and monocytes + untreated Tregs.

### 3.11. Assay of IL-10 and TGF-*β* in Supernatants from H37Rv-Infected Monocytes Cultured in the Presence and Absence of Non-Tregs and Tregs

**Figure 8:**
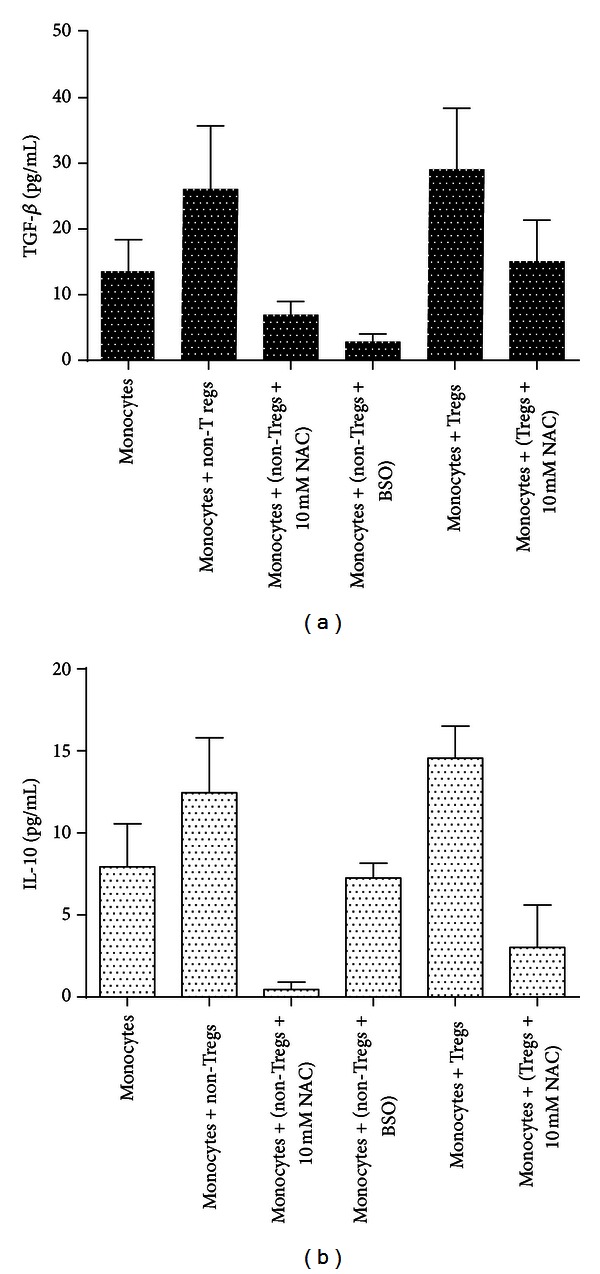
Assay of IL-10 and TGF-*β* in supernatants from monocytes + CD4+ T-cells. Cytokines in supernatants from monocyte Treg and monocyte-non-Treg co-cultures were measured on *n* = 5 samples by ELISA. Results were analyzed by ANOVA with Dunnett's multiple comparisons test, comparing all treatment categories to the monocyte only control, *P ≤ 0.05, **P ≤ 0.01. (a) TGF-*β*. (b) IL-10. Our results did not achieve statistical significance.

Results of our studies indicate that Tregs produce the most amounts of immunosuppressive cytokines such as IL-10 and TGF-*β* (Figures 8(a) and 8(b)) compared to monocytes and non-Tregs. Enhancing the levels of GSH in T cells (Tregs and non-Tregs) resulted in decreased synthesis of both TGF-*β* and IL-10 (Figures 8(a) and 8(b)) thereby augmenting the host responses to control *M. tb* infection.

## 4. Discussion

DCs are professional APCs that constitute 0.5%–1% of the leukocyte population in peripheral blood mononuclear cells. Majority of DC populations are present in nonlymphoid tissues and organs including skin, heart, liver, lung, and mucosal surfaces. DCs have the unique ability to link the innate and adaptive immune responses by initiating, stimulating, and regulating T cell responses, including antigen-specific T lymphocytes, Th1/Th2 modulation, Treg induction, and peripheral T cell deletion [12, 13].

DCs can either be myeloid derived DCs or lymphoid-derived DCs [14]. In this study myeloid derived DCs were generated *in vitro* by culturing peripheral blood monocytes in the presence of GM-CSF and IL-4 for 7 days [15]. 

 GSH has been previously reported as an antimycobacterial agent, capable of limiting the intracellular growth of *M. tb *in both murine and human macrophages. Thus, GSH has direct antimycobacterial activity and functions as an effector molecule in innate defense against *M. tb* infection [16–18]. Consistent with these observations, we have also found that GSH in combination with cytokines such as IL-2 and IL-12 enhances the activity of NK cells to control *M. tb* infection inside human monocytes [19]. Importantly, data from our most recent studies indicate that GSH activates the functions of T lymphocytes to control *M. tb* infection inside human monocytes [20]. Finally, we demonstrated that GSH levels are significantly compromised in macrophages, NK cells, and T cells isolated from individuals with HIV infection and this decrease correlated with several-fold increase in the intracellular survival of *M. tb* [5, 18–20]. All these observations support the fact the GSH controls *M. tb* infection by functioning as an antimycobacterial agent as well as by enhancing the functions of NK and T cells, and deficiency of GSH in immune cells derived from individuals with HIV infection is accompanied by diminished control of *M. tb* infection [5, 16–20].

 In this study we determined the effects of NAC (5 mM, 10 mM, and 20 mM) and L-GSH (5 *μ*M, 10 *μ*M, and 20 *μ*M) treatments in enhancing the levels of GSH in DCs thereby improving the control of intracellular *M. tb* infection. Although treatment of *M. tb*-infected DCs with NAC (20 mM) or L-GSH (20 *μ*M) resulted in maximum enhancement in the intracellular levels of *r*GSH (Figure 1(c)), we observed maximum inhibition in the growth of H37Rv when DCs were treated with either 10 mM NAC or 5 *μ*M L-GSH (Figure 2). These findings indicate that control of *M. tb* infection is achieved by supplying DCs with delicately balanced optimal concentrations of GSH. Interestingly, maximum inhibition in the growth of H37Rv was observed when DCs were treated with L-GSH at 5 *μ*M concentration (2000-fold lower concentration compared to NAC) (Figure 2). These results are consistent with our previous finding in macrophages, confirming that GSH functions as an effector molecule limiting the intracellular growth of *M. tb *inside DCs [16–18]. 

We observed that by enhancing the levels of GSH in DCs there is decreased production of IL-1 (Figure 3(a)). IL-1 is a proinflammatory cytokine and excess levels of IL-1 can cause fever, necrosis, and inflammation [5]. It is important to note that the concentrations of NAC/L-GSH that caused maximum reduction in the synthesis of IL-1 are the same concentrations that resulted in most effective inhibition in the intracellular growth of *M. tb* inside DCs (Figure 3(a)).

 During intracellular infections, DCs process the antigen, migrate to T cell rich lymph nodes, and present the peptides to T cells via MHC class molecules [21]. IL-12 secretion by DCs will induce naïve T cells to differentiate into Th1 (CD4+) subsets. Th1 cells produce IL-2 and IFN-*γ*. IFN-*γ* producing response is crucial for eliminating intracellular pathogens including *M. tb* [22]. Conversely, IL-10 secretion by DCs will induce naïve T cells to differentiate into Th2 subsets. Th2 cells are characterized by the production of IL-4 and IL-5 [22, 23]. These cytokines serve as growth and differentiation factors for B cells, respectively, leading to production of different classes of antibodies. Th2 responses are not considered useful for controlling intracellular infections [23].

 In our studies, we observed that increasing the levels of GSH in DCs by treatment with L-GSH (5 *μ*M) induced increased synthesis of IL-12, a cytokine that is responsible for polarizing CD4+ T cells to a Th1 subset (Figure 3(b)). This observation is consistent with the findings of other studies conducted which showed that treatment with lower concentration of NAC promotes IL-12 synthesis [24]. 

We found that enhancing the levels of GSH in DCs also resulted in decrease in the synthesis of IL-10 (Figure 3(c)). IL-10 is an immunosuppressive cytokine that can polarize the CD4 T cells to Th2 subtype. Therefore, decreased levels of IL-10 will indirectly favor Th1 CD4 T cell response. In contrast, conditions that will result in decreased levels of GSH, such as BSO-treatment, will enhance the synthesis of IL-10 leading to a Th2 CD4+ T cell response (Figure 3(c)). Th2 CD4+ T cell response will promote antibody production and will not result in control of *M. tb* infection and on the contrary will favor the intracellular growth of *M. tb*.

Immature DCs have high phagocytic and endocytic capabilities, and upon stimulation by microbial products or pro-inflammatory cytokines DCs mature into potent APCs. 

Upon maturation DCs express activating molecules CD83 or CMRF-44 and co-stimulatory molecules CD40, and B7-family members CD80 and CD86 [25]. DCs also upregulate their expression of HLA-DR and CD1, MHC class II surface receptors, used to process and present antigens to naïve T cells. 

DCs are sentinel cells surveying peripheral tissue as well as lymphoidal tissue for potential pathogens [10]. Upon recognition of a pathogen, DCs phagocytize pathogen and begin their maturation process where they reduce their phagocytic capabilities and begin their migration to the nearest lymph node. In the lymph node they present the pathogen to naïve T-cells via their MHC Class II molecule and co-stimulatory receptors of the B7 family, CD80, 86 and 40 [7]. 

Mature T cells are characterized by the expression of T cell receptor (TCR) and coreceptors CD4 and/or CD8 [22]. T cells express surface molecules such as CD28 molecule which interacts with DC cell surface molecules B7-1 (CD80) and B7-2 (CD86). Activation of T cells occurs when APCs, such as DCs, present pathogen peptides via major histocompatibility complex I or II (MHC class I or II) to TCR receptors; additionally, a co-stimulatory activation must occur between CD28 molecule and CD80 and CD86 on APCs, the CD28-CTLLA4 interaction [26]. 

Pathogens such as *M. tb* can inhibit complete maturation and expressions of co-stimulatory molecules that are necessary for migration into lymph nodes by inducing DCs to produce IL-10 [27]. Therefore, the key to *M. tb* control is to enhance macrophage and DC functions. 

 Results of our studies indicate that in contrast to untreated control DCs (Figures 4(a), 4(b), 4(c), 4(d), 4(e) and 4(f)) treatment with 10 mM NAC and 20 *μ*M L-GSH resulted in upregulation in the expressions of CD80, CD86 and HLA-DR on DCs that were infected *in vitro* with *M. tb *(Figures 4(a), 4(b), 4(c), 4(g), 4(h) and 4(i)). 

 LPS is a known inducer of DC maturation. Since NAC/L-GSH treatment of DCs not only improved the control of *M. tb* infection by its direct antimycobacterial effects but also upregulated the expressions of HLA-DR, CD80, and CD86, we tested whether treatment of DCs with a combination of NAC/L-GSH + LPS (1 *μ*g/mL) would result in further inhibition in the growth of intracellular *M. tb*. We tested our hypothesis by determining the intracellular survival of H37Rv in NAC/L-GSH + LPS treated DCs. Our results indicate that in comparison to other concentrations of NAC, treatment of DCs with 20 mM NAC in combination with LPS resulted in maximum inhibition in the growth of intracellular *M. tb* (Figure 5). Most importantly, treatment of H37Rv-infected DCs with a combination of LPS + L-GSH (20 *μ*M) resulted in complete stasis in the growth of H37Rv inside DCs (Figure 5). These results indicate that enhancing GSH in DCs in the presence of LPS results in complete stasis in the growth of H37Rv. Notably, L-GSH is able to induce this static effect (in conjunction with LPS) at a 1000-fold lower concentration compared to NAC (Figure 5). Our results indicate that the inhibition in the growth of *M. tb* inside GSH + LPS-treated DCs is due to combination of direct antimycobacterial effects of GSH and oxidative balance. Supplementing DCs with an L-GSH formulation provides complete *r*GSH molecules to cells, circumventing the enzymatic pathway responsible for *r*GSH production, without the requirement for the cell to construct the tripeptide. This may also explain why treatment with L-GSH seems to be more efficacious at much lower concentrations than NAC, as cells treated with NAC will have to produce new molecules of *r*GSH utilizing their own enzymatic machinery.

**Figure 9:**
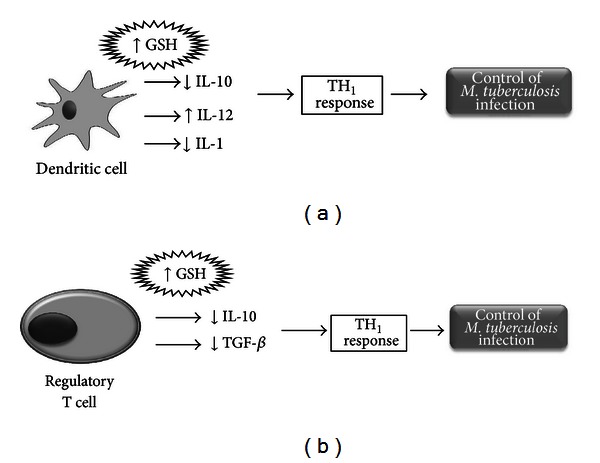
Model describing effects of GSH in growth control of *M. tb*. (a) Enhancing the levels of GSH in DCs decreases the synthesis of IL-10 polarizing the CD4 T cell response to a Th1 type. *M. tb* infection and conditions that will decrease the levels of GSH, such as BSO treatment will enhance the synthesis of IL-10 leading to a Th2 CD4+ T cell response. Th2 CD4+ T cell response will promote antibody production and will not result in control of *M. tb* infection and in contrast will favor the intracellular growth of *M. tb*. Enhancing the levels of GSH in DCs decreases the production of IL-1, a proinflammatory cytokine. Excess of IL-1 can cause fever, necrosis, and inflammation. Enhancing GSH in DCs induces increased synthesis of IL-12 thereby favoring a Th1 CD4+ T cell response. (b) Enhancing the levels of GSH in CD4+ T cells (regulatory and nonregulatory T cells) decreases the synthesis of TGF-*β* and IL-10 (immunosuppressive cytokines) thereby augmenting the host responses to control *M. tb* infection.

 We also tested the effects of GSH in enhancing the ability of DCs to induce proliferation of allogenic T cells by co-culturing allogeneic T cells with H37Rv-infected DCs. We observed that NAC treatment of DCs induced increased proliferation of allogeneic T cells (Figure 6(a)). Combination of NAC + LPS treatment did not result in further enhancement in the ability of DCs to induce proliferation of allogeneic T cells (Figure 6(a)). These results indicate that NAC treatment alone is sufficient for DCs to induce proliferation of T cells (Figure 6(a)). In separate studies, GSH levels were enhanced in allogeneic T cells (instead of DCs) by treatment with NAC and the effects of GSH supplementation in enhancing the proliferation of T cells in response to the H37Rv-infected DCs were tested. We observed that treatment of T cells with 5 mM NAC resulted in maximum proliferation when co-cultured with *M. tb*-infected DCs (Figure 6(b)). Our results confirm that GSH enhancement in DCs favor the control of *M. tb* infection. In addition, GSH enhancement upregulates the expressions of co-stimulatory molecules and IL-12, downregulates the synthesis of IL-1 and IL-10, and induces T cell proliferation (Figure 9(a)). However, these changes can occur only at optimum concentration of GSH and the concentration varies for each cytokine and marker, emphasizing the requirement of a fine balance in the levels of GSH to induce favorable changes.

 The main groups of T helper cell groups include Th1 cells, Th2 cells, Th17 cells, and Treg cells. Tregs are CD4+CD25+ T-cells which develop and emigrate from the thymus to perform their key role in immune homeostasis through the release of various cytokines [28]. Precise understanding of the immunosuppressive mechanism of Tregs remains elusive, although there is increasing evidence that Tregs manifest their function through various mechanisms that include the secretion of immunosuppressive soluble factors such as IL-10 and TGF-*β*, cell contact mediated regulation via the high affinity TCR and other co-stimulatory molecules such as CTLA-4, GITR, and cytolytic activity [29, 30]. We tested the intracellular survival of H37Rv inside monocytes co-cultured in the presence and absence of Tregs and non-Tregs. We observed an increased growth of H37Rv inside monocytes that were co-incubated with Tregs (Figure 7(b)). NAC treatment of Tregs resulted in improved control of H37Rv infection inside monocytes (Figure 7(b)). In contrast to monocytes that were co-incubated with Tregs, we observed a reduction in the fold growth of H37Rv when monocytes were cultured in the presence of non-T regs (Figure 7(a)). NAC treatment of non-T regs resulted in further reduction in the fold growth of H37Rv (Figure 7(a)). 

Our results also indicate that Tregs produce the highest amounts of immunosuppressive cytokines such as IL-10 and TGF-*β* (Figures 8(a) and 8(b)) compared to monocytes and non-Tregs. However, when we added NAC we observed a decrease in the amounts of IL-10 and TGF-*β* production (Figures 8(a) and 8(b)). Our results highlight the role of Tregs in favoring the growth of *M. tb* inside monocytes by producing immunosuppressive cytokines. Furthermore, decrease in IL-10 and TGF-*β* production following NAC treatment may mitigate the immunosuppressive effects of Tregs cells to allow for an effective immune response (Figure 9(b)) since studies have shown that increased TGF-*β* and IL-10 can directly suppress the Th1 response which is the first line of defense when battling intracellular infections [31]. Understanding the mechanisms by which Treg cells exert their influence is an area of continued intense research with broad implications for the development of therapeutic strategies for many disease processes including HIV and *M. tb* infection.

 Our research indicates that L-GSH and NAC are effective in modulating the levels of pro-inflammatory and immunosuppressive cytokines for beneficial innate and adaptive immune responses against *M. tb* (Figures 9(a) and 9(b)). Our long-term hope is for LGSH/NAC to be a future immune-adjunctive therapy for patients with refractory mycobacterial infections, especially for patients who are immunocompromised.
